# Mammalian fatty acid synthase: a commonly used viral host dependency factor and a putative target for host-targeted broad-spectrum antiviral therapeutic development

**DOI:** 10.1128/mbio.03954-24

**Published:** 2025-06-25

**Authors:** Paola N. Loperena González, Krithika P. Karthigeyan, Jacqueline Corry, Anurup Krishna, Ben Hackenberg, Beatriz Sierra, Jesse J. Kwiek

**Affiliations:** 1 Department of Microbiology and Center for Retrovirus Research, The Ohio State University 2647, Columbus, Ohio, USA; 2 Cellular, Molecular, and Biochemical Sciences Graduate Program, The Ohio State University 2647, Columbus, Ohio, USA; 3 Cellular Immunology Laboratory, Virology Department, Pedro Kourí Institute of Tropical Medicine (IPK) 115350, Havana, Cuba; Albert Einstein College of Medicine, Bronx, New York, USA

**Keywords:** virus-host interactions, FASN, antiviral pharmacology, flavivirus, coronavirus

## Abstract

Viruses regulate host processes to create cellular environments favorable to viral replication. At least 27 viruses that infect humans require host fatty acid synthase (FASN)-dependent *de novo* fatty acid biosynthesis, including viruses from the *Coronaviridae*, *Flaviviridae*, *Herpesviridae, Picornaviridae, Retroviridae,* and *Togaviridae* families. How could FASN activity and subsequent *de* novo fatty acid production impact viral replication? FASN activity produces the fatty acid palmitate, which can be further metabolized into fatty acids that are used to form lipid droplets that can be used during viral assembly and budding, for beta-oxidation to generate ATP, and to create fatty acyl groups used for post-translational protein modification to change the subcellular localization of viral or host proteins. In this minireview, we outline the function of FASN, review the mechanisms linking virus replication and fatty acid biosynthesis, and consider the potential of FASN as a target for broad-spectrum antiviral drug development.

## INTRODUCTION

Fatty acid synthase (FASN) is a metabolic enzyme that catalyzes *de novo* fatty acid synthesis. In healthy adults, FASN is expressed at very low levels in most tissues because most normal tissues and cells obtain fatty acids exogenously from their diet (1). FASN expression is highly regulated in cells and can change 10-fold (or more) in response to stresses such as starvation, lactation, or pathological states (2). Viral infection has also been linked to changes in FASN expression, and inhibition of human FASN activity can attenuate the replication of at least 27 viruses from 15 different families (Table 1). Because viruses are obligate intracellular parasites that cannot replicate without the help of host cellular machinery and because FASN is required by so many viruses, it is plausible that FASN could be a target for broad-spectrum antiviral therapeutic development. Drugging host proteins could provide several advantages over traditional targeting of viral targets, including a higher barrier to drug resistance (human proteins do not evolve as rapidly as viral proteins), and the potential development of a pan-antiviral (if several viruses require the same host pathway). The challenge is to identify a druggable host pathway that is also critical for viral replication. In the next section, we review the cellular regulation and mechanism of FASN and discuss how viruses take advantage of its enzymatic function during infection.

**TABLE 1:** Viruses that depend on mammalian FASN for infection

Family	Virus	Effect of direct FASN inhibition on viral replication *in vitro^a^*	Reference(s)
*Astroviridae*	Astroviruses	Reduced infectious virion production, synthesis of antigenomic RNA, and structural VP90 protein levels	Murillo (3)
*Bunyaviridae*	Rift Valley fever virus (RVFV)	Reduced intracellular nucleocapsid levels	Moser (4)
*Coronaviridae*	Severe acute respiratory syndrome coronavirus 2 (SARS-CoV-2)	Reduced infection, viral RNA genomic levels, and infectious virion production of wild-type and variants of concern (alpha, beta, gamma, and delta variants) and Spike glycoprotein palmitoylation; *in vivo* (mice), reduced viral RNA and infectious viral titers, improved mice survival, and reduced infection, inflammation, and lesion size in lung tissues	Chu (5), Aliyari (6), Farley (7), Li (8)
	Middle Eastern respiratory syndrome coronavirus (MERS-CoV)	Reduced infectious virion production	Yuan (9)
*Flaviviridae*	Hepatitis C virus (HCV)	Reduced HCV replication, HCV protein levels, viral RNA synthesis, and virion production in both HCV infection and HCV replicon systems	Huang (10), Yang (11), Nasheri (12)
	Dengue virus (DENV)	Reduced viral infection and virion production	Samsa (13), Heaton (14), Tongluan (15)
	West Nile virus (WNV)	Reduced viral RNA synthesis and virion production	Heaton (14), Martin-Acebes (16)
	Yellow fever virus (YFV)	Reduced infectious virion production	Heaton (14)
Hepadnaviridae	Hepatitis B virus (HBV)	Reduced virion production and accumulated intracellular viral nucleocapsid proteins	Okamura (17)
Herpesviridae	Herpes simplex virus-1 (HSV-1)	Reduced infectious virion production	Aliyari (6)
	Varicella-zoster virus (VSV)	Inhibited viral replication and maturation of viral glycoproteins	Namazue (18)
	Human cytomegalovirus (HCMV)	Reduced infectious virion production	Munger (19)
	Epstein-Barr virus (EBV)	Reduced EBV early viral protein levels, viral LMP1 induced FASN protein expression, and primary B-cell transformation	Li (20), Hulse (21)
	Karposi sarcoma herpesvirus (KSHV)	Induced apoptosis and death of infected cells	Delgado (22)
*Orthomyxoviridae*	Influenza virus	Reduced infectious virion production	Munger (19)
*Paramyxoviridae*	Human parainfluenza virus 3 (PIV3)	Reduced infectious virion production	Ohol (23)
*Picornaviridae*	Coxsackievirus B3 (CVB3)	Reduced infectious virion production, viral RNA, and protein synthesis	Rassmann (24), Wilsky (25)
	Enterovirus A71	Reduced infectious virion production	Yang (26)
	Rhinovirus	Reduced infectious virion production	Nguyen (27), Ohol (23)
Poxviridae	Vaccinia virus	Reduced viral protein synthesis, viral assembly, and caused accumulation of intracellular viral DNA	Greseth (28)
*Pneumoviridae*	Respiratory syncytial virus (RSV)	Reduced the production of infectious RSV A and B strain virions, viral RNA and protein levels, viral spread, and infectivity; reduced infection in mice *in vivo*	Ohol (23)
*Reoviridae*	Rotavirus	Reduced viral infectivity and RNA levels *in vitro*	Gaunt (29)
*Retroviridae*	Human immunodeficiency virus type-1 (HIV-1)	Inhibited myristoylation and proteolytic cleavage of HIV-1 Gag to p24, reduced virion production, prevented myristoylation of ectopic HIV-1 Matrix (p17)	Pal (30), Kulkarni (31), Karthigeyan (32)
*Rhabdoviridae*	Vesicular stomatitis virus (VSV)	Reduced progeny virion production	Aliyari (6)
*Togaviridae*	Chikungunya virus (CHIKV)	Reduced viral genome replication and RNA levels and ectopic NSP1 palmitoylation	Zhang (33), Bakhache (34)
	Mayaro virus (MAYV)	Reduced viral replication	Bakhache (34)
	Semliki Forest virus (SFV)	Reduced viral replication	Royle (35)

^*a*^ FASN inhibition is described *in vitro,* unless otherwise stated as *in vivo*.

## FASN BIOCHEMISTRY AND REGULATION

FASN is a 273 kDa, cytoplasmic, homodimeric enzyme that uses NADPH to condense acetyl-CoA and malonyl-CoA into the 16-carbon fatty acid palmitate (C16:0) (36). FASN uses multiple catalytic domains to synthesize palmitate, starting with acetyl-CoA, which is elongated two carbons per cycle via the addition of malonyl-CoA (36). The catalytic domains of FASN include malonyl-acetyl transferase (MAT), β-ketoacyl synthase (KS), β-ketoacyl reductase (KR), dehydratase (DH), and enoyl reductase (ER); movement of the elongating fatty acid chain through these domains is mediated by the acyl carrier protein (ACP), and once formed, palmitate is released from FASN by the thioesterase (TE) domain. Nascent palmitate can be shortened (e.g., myristate [C14:0]), elongated (e.g., stearate [C18:0]), and desaturated (e.g., oleate [C18:1]) to form a diversity of fatty acids. Newly synthesized fatty acids contribute to cellular energy homeostasis, complex lipid formation (e.g., phospholipids, sphingolipids, and triacylglycerols), and protein fatty acylation for the regulation of subcellular localization and membrane association (for review, see references 37–39).

Because mammalian cells can acquire fatty acids from dietary sources (40, 41), FASN is not highly expressed in most tissues, with the exception of the liver, adipose tissue, and lactating mammary glands (42). In contrast to its expression in most normal tissues, FASN is overexpressed in several cancers (43–47), diabetes (48, 49), obesity (49), and non-alcoholic fatty liver disease (NAFLD) (50, 51). FASN transcription is regulated by several transcription factors, including sterol regulatory element binding protein 1 (SREBP-1), liver X receptors (LXRs) (51, 52; for review, see reference 53), and carbohydrate response element binding protein (ChREBP) (39). These transcription factors are normally induced by hormonal (e.g., insulin) or dietary compounds (e.g., carbohydrates, oxysterols) that ultimately lead to FASN protein expression (51, 52). In addition to transcriptional regulation, FASN protein levels are also regulated by post-translational modifications, including phosphorylation and ubiquitination, as documented in breast cancer cells (54), and obese mice (55), respectively. Finally, Type-I interferon (IFN-1), an antiviral host factor, can downregulate FASN protein levels, perhaps as an antiviral host defense mechanism (6).

## FASN AND VIRAL INFECTION

**Fig 1:**
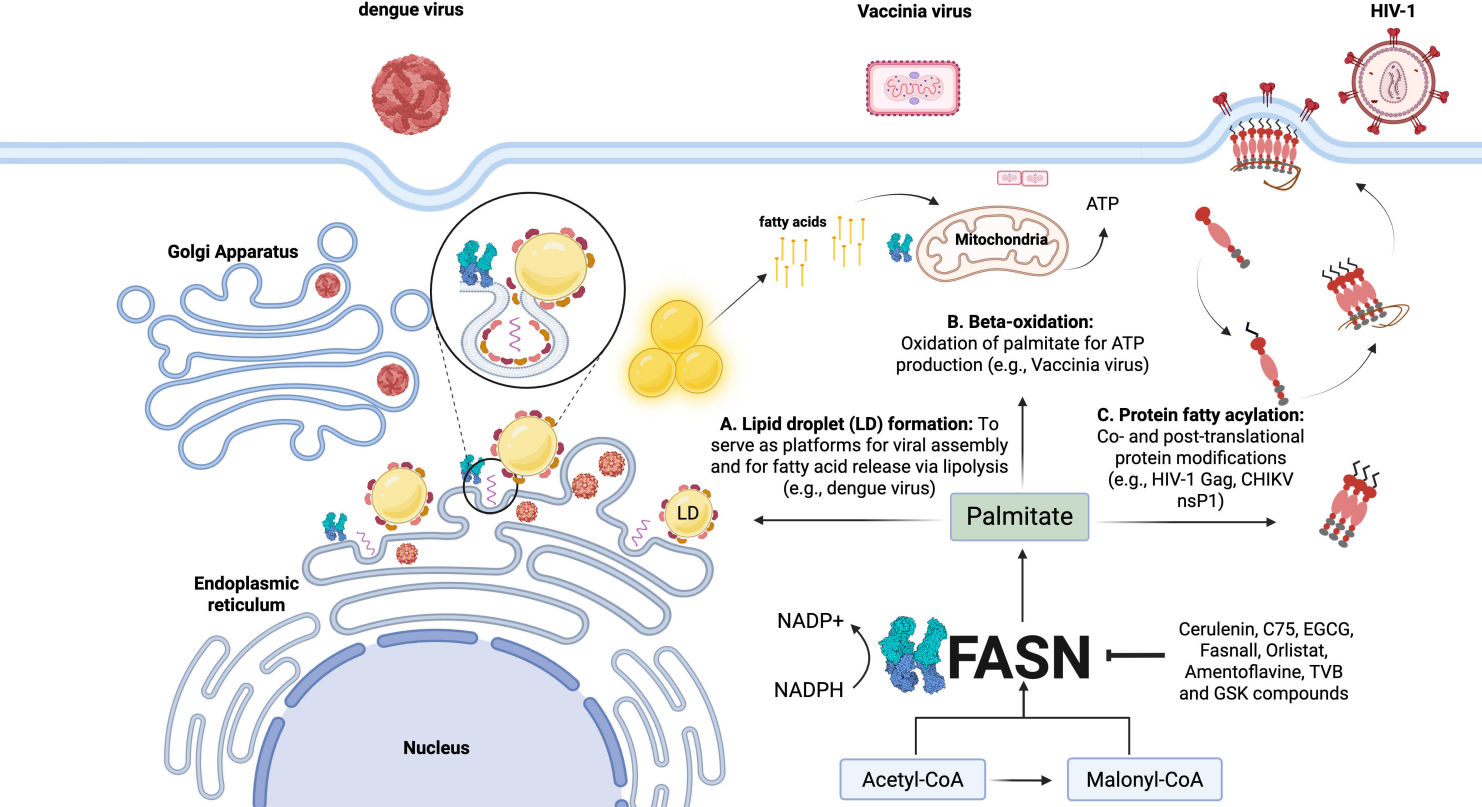
FASN and viral replication. FASN is implicated as a host-dependency factor in at least 27 viruses of concern to humans. Three main mechanisms link FASN-dependent fatty acid biosynthesis to viral infection, including the formation of lipid droplets, mitochondrial beta-oxidation, and host or viral protein fatty acylation. (**A**) During infection, lipid droplets can serve as platforms for viral proteins (e.g., capsid proteins) to facilitate viral particle assembly within replication complexes. (**B**) Additionally, lipid droplets are lysed to release fatty acids used for mitochondrial beta-oxidation for ATP production (e.g., dengue virus). FASN-derived palmitate is also transported into the mitochondria for oxidation to generate an energetically favorable environment for viral replication (e.g., Vaccinia virus). (**C**) Palmitate and its beta-oxidized product myristate can function as co- and post-translational protein modifications, respectively, to regulate protein subcellular localization. For example, the major structural HIV-1 polyprotein, Gag, is myristoylated in a FASN-dependent manner to promote Gag localization to the plasma membrane and particle assembly. The FASN dependency of various viral families makes FASN an intriguing target for broad-spectrum antiviral development. Figure and FASN structure (PDB: 2VZ9) generated with BioRender. The mechanism of dengue virus assembly on lipid droplets is inspired by and adapted from Zhang et al. (33).

In addition to the well-documented FASN upregulation associated with cancer and metabolic diseases, viral infections can also increase intracellular FASN levels, and many viruses require FASN activity to replicate (Table 1). How could FASN activity and subsequent *de novo* fatty acid production affect viral infection? The product of FASN, palmitate, and its derived fatty acid metabolites has cellular functions that could benefit viral replication, including (i) the formation of lipid droplets that can anchor viral replication complexes or generate energy, (ii) energy production via mitochondrial beta-oxidation, and (iii) post-translational protein modification by fatty acylation (Fig. 1).

Viral infection can induce intracellular membrane rearrangements within organelles (e.g.*,* endoplasmic reticulum) to enable the formation of viral replication complexes (for review, see reference 56). In some cases, viral replication induces lipid droplets to form in close apposition to viral replication complexes. Lipid droplets are cytoplasmic storage organelles derived from the endoplasmic reticulum, and lipid droplets comprise a hydrophobic core of neutral lipids (triacylglycerols) surrounded by a monolayer of phospholipids and proteins (for review, see references 57, 58). In cells, lipid droplets store excess lipids, maintain lipid homeostasis, and respond to cellular stress (57, 58). In viral replication, lipid droplets can have many functions, including to provide a scaffold for RNA replication as well as viral particle assembly and maturation through association with viral capsids (13, 59); to contain lipid resources, like fatty acids and phospholipids, that viruses can use to acquire their viral membranes; and finally, to contribute to mitochondrial fatty acid oxidation via lipolysis (for review, see references 60, 61). Interestingly, FASN inhibition can reduce both the number and size of lipid droplets induced by viral infection, demonstrating an association between FASN activity and the formation of lipid droplets during infection (13, 26). Viruses from the *Flaviviridae* (13, 62–66) and *Coronaviridae* (7, 9, 67) families have been shown to require lipid droplets for replication.

Viral infection requires an energetically favorable cell environment to replicate the genome and produce infectious virions (68). Mitochondrial fatty acid oxidation, also known as beta-oxidation, is the primary fatty acid degradation pathway, and it produces energy in the form of ATP (69). In this process, long-chain fatty acids are transported into the mitochondria and converted to fatty acyl carnitine molecules by carnitine:palmitoyltransferase 1 (CPT1), then they are shortened in the mitochondrial matrix to yield NADH, FADH_2_, and acetyl-CoA. Beta-oxidation-derived acetyl-CoA can enter the tricarboxylic acid cycle (TCA) to generate ATP via oxidative phosphorylation (26, 62), whereas NADH and FADH_2_ molecules can directly enter the oxidative phosphorylation pathway to generate ATP (70). Some viral infections, like dengue virus (62) and enterovirus-A71 (26), lyse lipid droplets to supply fatty acids for beta-oxidation. In normal cells, fatty acid synthesis and fatty acid oxidation are antagonistic processes that are highly regulated to minimize co-occurrence; in cancer cells, both processes can occur simultaneously as observed with isotope labeling of citrate in skin cancer cells (71). In viral infection, the balance between fatty acid synthesis and fatty acid oxidation remains unknown. Viruses such as dengue virus (62), enterovirus -A7 (26), and vaccinia virus (28) replication have been shown to require ATP generation via β-oxidation.

Another way that FASN activity could contribute to viral replication is via the generation of fatty acids used for protein fatty acylation, which is the covalent attachment of fatty acids to proteins. Protein fatty acylation includes palmitoylation and myristoylation, and these modifications increase protein hydrophobicity, which can alter a protein’s subcellular localization, intracellular trafficking, protein-protein interactions, and membrane binding (72). Protein palmitoylation is one of the most common post-translational lipid modifications, and it occurs when a palmitoyl transferase attaches palmitate to a cysteine residue (38, 72). Protein myristoylation is less common than protein palmitoylation and occurs when N-myristoyl transferase attaches myristate co-translationally to an N-terminal glycine residue (for review, see references 38, 72). Viral proteins such as coronavirus spike glycoprotein, chikungunya virus nsP1 protein, and HIV-1 Gag can be fatty acylated in a FASN-dependent manner to facilitate plasma membrane binding and subsequent viral assembly and budding (8, 9, 32, 34, 73–81).

In addition to lipid droplet formation, ATP generation, and protein fatty acylation, FASN-generated palmitate and its derivatives can form the hydrophobic tail of complex lipids, which can be used to generate and maintain structural membranes and lipid droplets in cells (38, 53). Although it has not been experimentally demonstrated as a FASN-dependent process, viruses could require FASN to produce the complex lipids that form viral envelope membranes or to alter the cellular membrane composition for viral replication. For example, many viruses use lipid rafts for viral assembly and budding, including human immunodeficiency virus type 1 (HIV-1) (82) and respiratory syncytial virus (RSV) (83). Lipid rafts are microdomains in cellular membranes, composed mostly of glycosphingolipids and cholesterol, that compartmentalize cellular processes like signal transduction and vesicle trafficking. In infected cells, lipid rafts serve as entry and/or viral assembly platforms for budding and infectious virion production (84). Although currently, there is no evidence linking FASN activity to lipid raft formation during viral infection, FASN activity has been linked to both phospholipid production and lipid raft generation in epithelial cancer cells (85).

## PHARMACOLOGICAL FASN INHIBITION AND THE EFFECTS ON CELL METABOLISM

Several small molecules can inhibit FASN activity, including C75, cerulenin, amentoflavone, epigallocatechin-3-gallate (EGCG), Fasnall, GSK2194069, GSK1995010, Orlistat, and a family of “TVB” inhibitors (for review of FASN inhibitors, see references 86, 87). C75 (88) is a synthetic FASN inhibitor related to the natural product cerulenin, a fungal antibiotic isolated from *Cephalosporium caernlens,* and both inhibit FASN β-ketoacyl synthase activity (89). In addition to β-ketoacyl synthase, C75 also irreversibly inhibits FASN enoyl reductase and thioesterase activities (90). However, C75 has off-target effects by acting as a CPT1 agonist and increasing fatty acid oxidation and ATP levels, leading to profound weight loss and anorexia in animal models (91). Orlistat (92) is an FDA-approved anti-obesity drug that targets the FASN thioesterase activity, although it also irreversibly inhibits pancreatic and gastric lipases (93). TVB-2640 (denifanstat), which inhibits FASN β-ketoacyl reductase activity, is the most clinically advanced FASN inhibitor (94), and it is currently in clinical trials for the treatment of metabolic disorders, several cancers, and dermatology indications. Two compounds related to TVB-2640**,** TVB-3166, and TVB-3664 have demonstrated *in vitro* anti-cancer activity as well (45, 95, 96). Importantly, denifanstat has shown a favorable safety profile (94, 97, 98), indicating that FASN is a druggable host target.

A metabolic consequence of FASN inhibition is a buildup of malonyl-CoA, a known allosteric inhibitor of CPT1, which, when inhibited, reduces mitochondrial fatty acid oxidation (91). Since cancerous cells require *de novo* lipogenesis and fatty acid oxidation to continuously proliferate, CPT1 allosteric inhibition induces a cytotoxic effect, likely through ceramide buildup, that leads to apoptosis (99). This effect is not seen in normal cells as their *de novo* lipogenesis is at low levels, suggesting that the toxic effects of FASN inhibition are selective to cancerous cells (45). In contrast, mice fed with a high-fat diet and treated with TVB-3166 do not accumulate malonyl-CoA but rather have increased malonylation of the proteome, suggesting a mechanism to buffer the unused malonyl-CoA that was accumulated (100). Thus, malonyl-CoA buildup due to FASN inhibition might be a toxic trait for cancer cells only; however, the effects of malonyl-CoA buildup during viral infection remain unknown. In addition to the effects on malonyl-CoA levels, pharmacological FASN inhibition in cancerous cell lines can lead to other metabolic consequences, including increased levels of glycerol, glycerophospholipids, diacylglycerols, polyunsaturated fatty acids, and lipid droplet accumulation (45, 99, 100). These changes are not universal, as FASN inhibition did not affect the fatty acid levels in organs with lower FASN activity (e.g., heart), reinforcing the heterogeneity of FASN activity in pathological states (100). Similar to the in-depth studies of the FASN signalome in the field of oncology, further studies should focus on understanding how viral infection affects the FASN signalome (101).

FASN-catalyzed *de novo* fatty acid synthesis and its byproducts also play a role in immune activation. In macrophages, FASN activity contributed to the production of acetoacetyl-CoA, a building block for cholesterol synthesis (102). Acetoacetyl-CoA is formed when acetyl-CoA and malonyl-CoA are condensed in the beta-ketoacyl synthase (KS) domain. FASN inhibition with C75, which inhibits FASN KS activity, prevented the production of acetoacetyl-CoA and disrupted an LPS-stimulated pro-inflammatory response by preventing the translocation of Toll-like receptor 4 (TLR-4) to lipid rafts in macrophages. Interestingly, FASN inhibitors that function downstream of KS (e.g.,GSK2194069 and Orlistat) do not affect LPS-mediated pro-inflammatory response, suggesting that acetoacetyl-CoA synthesis was not abrogated. Thus, the FASN requirement for a pro-inflammatory response in macrophages is essential but can be mitigated depending on the FASN domain targeted (103). As FASN is important for immune activation (for review, see reference 104), future studies should elucidate if an immune response occurs upon FASN inhibition to support the clearing of viral infections.

## ANTIVIRAL THERAPEUTICS

Despite the massive amount of morbidity and mortality caused by viruses, the vast majority of the existing antivirals can be used to treat only a few of the known human viral pathogens (105). Because disparate viruses and virus families encode different enzymes, current (pathogen targeted) antiviral treatment is often described as “one bug, one drug,” meaning each virus needs its own specific drug to target specific viral proteins. As a result, the vast majority of antiviral therapeutics target unique viral proteins, although approximately 10% of all approved antivirals are host-directed, with more than half of these targeting interferon signaling (105). Other than ribavirin (106, 107), there are few antiviral therapeutics capable of effectively treating multiple existing or emerging viral pathogens. Because all viruses use host proteins and pathways to replicate, there are thousands of host proteins required for viral infection (108–111). As we describe herein, some proteins, such as human FASN, are commonly used by multiple viruses, and they have the potential to become a broad-spectrum antiviral therapeutic target. Next, we review the specific mechanisms linking viral replication to FASN-dependent *de novo* fatty acid biosynthesis, arranged by the virus family.

### Flaviviridae

Members of the *Flaviviridae* family are enveloped, positive-sense single-stranded RNA viruses that include dengue virus (DENV), West Nile virus, Zika virus, and hepatitis C virus. DENV spreads through the bite of the *Aedes* mosquito, and it comprises four serotypes (DENV 1-4) that can cause a range of conditions from flu-like symptoms to severe dengue hemorrhagic fever or dengue shock syndrome (112). DENV-2 replication was reduced by the treatment of cells with FASN-specific siRNA, cerulenin, or C75, and Orlistat treatment significantly reduced infection and virion production of a lab-adapted DENV-4 strain and clinical hemorrhagic fever DENV-4 isolate (13–15). In cells, DENV infection rearranges the endoplasmic reticulum membrane to form replication complexes that allow for efficient genome replication and viral encapsidation (113). It has been shown that DENV infection relocalizes FASN to active replication complexes to promote *de novo* fatty acid biosynthesis. Labeling of DENV-infected cells with ^14^C-acetate or ^14^C-malonyl-CoA indicates that compared with uninfected cells, DENV-infected cells have increased *de novo* synthesized fatty acids, specifically in subcellular compartments containing DENV RNA (14). Despite its requirement during infection, FASN protein levels do not dramatically increase during DENV infection (14, 15), perhaps because during DENV infection, FASN protein localization is manipulated by DENV non-structural protein 3 (NS3) (14, 114). Ectopically expressed DENV NS3 interacted with FASN at the endoplasmic reticulum, and increased NS3 expression levels correlated with increased FASN activity, in a dose-dependent manner. FASN-NS3 complex recruitment to viral replication complexes in the endoplasmic reticulum is mediated by Rab-18, a GTPase involved in vesicle trafficking (114), and in cells with functional Rab-18, DENV-2 infection caused FASN to colocalize to the endoplasmic reticulum and induced lipid droplet formation. This suggests that *in vitro,* DENV-2 NS3 interaction with FASN at sites of DENV replication can stimulate FASN *de novo* fatty acid biosynthesis (14).

At replication complexes, lipid droplets serve as assembly platforms for DENV capsid protein where they accumulate on the surface of the lipid droplets to promote viral encapsidation (13, 59). Mutations in DENV-2 capsid that prevent lipid droplet targeting or mutations that cause capsid dissociation from lipid droplets are associated with defects in viral particle production, but not in viral RNA translation or synthesis (13, 59). Thus, lipid droplets formed during DENV infection could provide an assembly platform for viral encapsidation on their surface. Another role for lipid droplets during DENV infection is the provision of fatty acids for beta-oxidation and ATP production (13, 62). Lipophagy, or autophagy of lipid droplets for lysis (62), increased beta-oxidation in DENV-2-infected cells, and treatment of DENV-infected cells with etomoxir, a CPT1 inhibitor that blocks mitochondrial import of fatty acids for beta-oxidation, decreased DENV RNA replication and release of progeny virions (62). Given that research groups have observed both decreased and increased lipid droplet formation during DENV infection, it is possible that DENV infection increases fatty acid synthesis, which leads to lipid droplet formation in the service of viral assembly; subsequently, these lipid droplets can be used for ATP production to generate an energetically favorable environment. This role of FASN during DENV infection stems from the observation that C75 treatment reduced lipid droplet accumulation and DENV replication in infected cells (13); however, since C75 also increases CPT1 activity, the observed reduction of lipid droplets could be an indirect consequence of FASN inhibition. Further research should explore the connection between FASN and lipid droplet formation in DENV infection with FASN-specific inhibitors or other methods to degrade FASN (reviewed in reference 101).

West Nile virus (WNV) and Zika virus (ZIKV) belong to the same genus (e.g., *Flavivirus*) as DENV. WNV is an arthropod-borne virus that primarily infects birds, although humans can be infected and lead to serious neurological diseases (115)*. In vitro*, FASN localizes to WNV replication complexes early after infection, and like DENV infection, FASN localization was not accompanied by changes in FASN protein levels. FASN inhibition by cerulenin or C75 reduced WNV viral RNA synthesis and virion production (16).

ZIKV is another arthropod-borne virus that has been linked to birth defects (116), especially when ZIKV infection occurs during the first trimester of pregnancy (for review, see reference 116). *Ex vivo*, ZIKV infection of first trimester placental explants increased levels of *fasn* mRNA, phospholipids, diacylglycerols, triacylglycerols, and total neutral lipids (117), and it also increased both the size and amount of lipid droplets. Lipid droplets are production sites of inflammatory bioactive lipid molecules (57, 58), and many bioactive lipid molecules increased in ZIKV-infected placental explants. ZIKV infection of neuroblastoma cells increased *fasn* mRNA levels and intracellular lipid droplet content (67), and several groups have reported that ZIKV capsid protein both colocalizes and interacts with lipid droplets (63, 64). Similarly, ZIKV infection in human neural stem cells increased SREBP-1 and lipid droplet amounts in human neural stem cells, and pharmacological inhibition of lipid droplet formation in these cells reduced ZIKV replication (67). Likewise, inhibition of lipid droplet formation in an acute ZIKV infection mouse model reduced the ZIKV viral load and increased the newborn mouse survival rate (67*). In vitro*, cerulenin treatment of ZIKV-infected A549 cells, a human non-small lung cancer cell line, was not shown to reduce ZIKV replication, even at varying virus titers and cerulenin concentration, which the authors speculated could be attributed to increased FASN levels (35). However, in an African green monkey kidney epithelial cell line (Vero cells), treatment with AM580, an inhibitor of the transcription factor SREBP-1, inhibited ZIKV replication (9). Although the requirement for FASN remains unresolved, ZIKV infection increases *fasn* mRNA levels, likely through transcriptional activation, and promotes the formation of lipid droplets (9, 67).

Hepatitis C virus (HCV) is a bloodborne pathogen that belongs to a distinct genus in the *Flaviviridae* family (e.g., *Hepacivirus*), and HCV infection is associated with chronic hepatitis, which can develop into cirrhosis and hepatocellular carcinoma (10). The requirement for FASN during HCV infection is highlighted when in hepatocellular cell lines, FASN inhibition by short hairpin-based interfering RNA (11, 12), siRNA (10), C75 (10–12), Orlistat (10–12), or cerulenin (12) reduced HCV replication, protein levels, viral RNA synthesis, and virion production in both HCV infection or HCV replicon systems (10–12). HCV infection and viral proteins induced the upregulation of FASN levels both *in vitro* (10–12) and *in vivo,* likely through transcriptional activation. Although HCV infection did not change FASN subcellular localization (12), FASN both colocalized and interacted with HCV NS5B, the viral RNA-dependent RNA polymerase (10). *In vitro*, FASN retained NS5B in active replication complexes at the endoplasmic reticulum, increasing its RNA polymerase activity (10). HCV NS5A also interacted with FASN in replicon cell systems, but this interaction required NS5B, suggesting that FASN interacts with NS5A through NS5B.

*In vivo*, in the chimeric SCID/Alb-uPA mouse model, HCV infection increased hepatic FASN protein levels and activity (12). Another *in vivo* study reported that lentiviral expression of NS5A protein in mice increased *SREBP1* mRNA, both *fasn* mRNA and protein levels, serum levels of free fatty acids, triglycerides, and liver tissue lipid droplet accumulation (118). Similar results are reported *in vitro*, where mouse and human hepatoma cell lines expressing NS5A increased both *srebp1* and *fasn* mRNA and protein levels and had lipid droplet accumulation (118). HCV core protein also increased *srebp1* and *fasn* mRNA and protein levels, increased neutral lipid staining (65), and was found to localize to lipid droplets (66). A proteomic and lipidomic analysis of HCV-infected cells revealed that infection increased FASN and lipogenic enzymes involved in peroxisomal and mitochondrial fatty acid oxidation. Finally, viral protein fatty acylation could also provide a mechanistic link between FASN and HCV replication. HCV core protein palmitoylation is required for virus assembly and efficient release of progeny (74). Core protein palmitoylation causes lipid droplet accumulation in HCV-infected cells, and it is required for a core protein to associate with the endoplasmic reticulum, in close apposition to lipid droplets (74). Furthermore, NS2 palmitoylation is required for HCV RNA replication and virus particle assembly (75), and abrogation of NS2 palmitoylation impairs envelope protein 2 (E2) recruitment to virus assembly sites. Thus, FASN could contribute to HCV replication by providing fatty acids to form lipid droplets that serve as viral assembly platforms. It could fuel beta-oxidation, and it could also contribute to post-translational modification of HCV proteins.

### Coronaviridae

Viruses in the *Coronaviridae* family are enveloped, positive-sense RNA viruses that include betacoronaviruses like severe acute respiratory syndrome coronavirus 2 (SARS-CoV-2) and Middle East respiratory syndrome coronavirus (MERS-CoV). SARS-CoV-2 is the causative agent of COVID-19, a respiratory disease with high fatality rates, especially in the elderly, immunosuppressed, and those with comorbidities (119). *In vitro,* FASN inhibition via shRNA (5), gene knockout (5), or Orlistat, TVB-2640, TVB-3664, TVB-3166, C75, EGCG, Cerulenin, or GSK2194069 (5–7) significantly reduced SARS-CoV-2 infection, viral RNA levels, and infectious virion production. One FASN inhibitor, TVB-3664, was five times more potent at blocking SARS-CoV-2 replication *in vitro* than remdesivir, an FDA-approved anti-viral nucleoside analog (5). *In vivo,* daily Orlistat treatment of mice reduced inflammation and SARS-CoV-2 replication and improved animal survival.

Although a causal link between FASN and lipid droplet formation during coronavirus infection has not been reported, lipid droplets are important for coronavirus infection. *In vitro*, SARS-CoV-2 infection increased triacylglycerol levels and both the number and the size of lipid droplets (7). Pharmacological inhibition of lipid droplet formation decreased SARS-CoV-2 viral replication and prevented cell death in infected primary monocytes (120). Additionally, SREBP-1 expression was increased in SARS-CoV-2-infected human primary monocytes (120), and SREBP-1 inhibition by AM580 treatment reduced the levels of both viral RNA and viral proteins in various SARS-CoV-2-infected cell lines (121). Two groups identified lipid droplets in SARS-CoV-2-infected cells (7, 120), and one group reported viral particles and viral double-stranded RNA in close apposition to lipid droplets (120), where it was observed that fully assembled viral particles associated with the phospholipid monolayer of lipid droplets (120). Additionally, treating SARS-CoV-2-infected cells with Orlistat caused the accumulation of lipid droplets, which reduced virus replication (7). It is possible that SARS-CoV-2 replication, like DENV replication, requires a balance of lipid droplet formation and breakdown, to support both viral assembly and provide fatty acids for ATP production. Additional studies are needed to better define the mechanisms that link FASN, lipid droplets, and SARS-CoV-2 replication.

MERS-CoV causes a range of symptoms from mild upper respiratory symptoms to severe pneumonia to multi-system failure (for review, see reference 122). MERS-CoV infection of an *in vitro* human bronchial epithelial cell line increased *fasn* mRNA levels and altered the cellular lipid profile, including changes in several fatty acids and complex lipids like glycerophospholipids, and treatment of MERS-CoV-infected cells with C75 reduced infectious virion production. Furthermore, coronavirus spike glycoprotein is post-transcriptionally modified with palmitate, which aids in cell-cell fusion, viral entry, and virion infectivity, and FASN inhibition with C75 reduced spike glycoprotein palmitoylation and restricted SARS-CoV-2 spread *in vitro* (8, 9, 73). Lipid droplets also accumulate during MERS-CoV-2 infection, in an SREBP-1-dependent manner, as AM580 treatment reduced infectious virion production, viral nucleoprotein levels, accumulation of lipid droplets, and formation of double-membrane vesicles *in vitro* (9). *In vivo*, AM580 treatment of mice inhibited MERS-CoV infection and reduced MERS-CoV-associated pneumonia and encephalitis. These findings show the importance of *de novo* lipogenesis in the modulation of coronavirus infection.

### Picornaviridae

Members of the *Picornaviridae* family are non-enveloped, positive-sense, single-stranded RNA viruses that include human pathogens like enteroviruses, coxsackieviruses, and rhinoviruses that can cause a wide range of illnesses. Enterovirus A71 (EV-A71) is one of the causative agents of hand, foot, and mouth disease, whose complications can lead to neurological disorders (123). Human umbilical vein endothelial cells infected with EV-A71 increased lipid droplet accumulation, and C75 treatment of infected cells decreased lipid droplet size, increased cell viability, and reduced infectious virion production. Inhibition of beta-oxidation with etomoxir, an irreversible inhibitor of CPT1, reduced EV-A71 mRNA levels and viral titers (26). Furthermore, knockdown of the CPT1 gene reduced viral replication, highlighting the importance of beta-oxidation in EV-A71 infection (26). Even after treatment with C75, which can also affect CPT1 activity, EV-A71 infection was reduced, suggesting that *de novo* fatty acid synthesis from FASN activity is essential for productive viral infection. Thus, *in vitro*, during Enterovirus A71 infection, FASN activity contributes to lipid droplet formation, likely for energy production.

Coxsackievirus B3 (CVB3) causes acute and chronic infections and is associated with severe complications to heart function and various other conditions (for review, see reference 124). *In vitro*, CVB3 upregulated FASN expression in a B-lymphocyte cell line (25), and in HeLa cells, FASN inhibition with cerulenin or C75 reduced CVB3 replication and production of infectious virions while delaying infection-induced cytopathic effects (24). Orlistat and amentoflavone treatment (125) reduced CBV3 replication, and amentoflavone reduced viral RNA and protein synthesis, although it did not reduce *fasn* mRNA and protein levels (25).

Rhinoviruses (RV) are referred to as common-cold viruses; however, in patients with pneumonia, asthma, or other pulmonary diseases, RV infection can lead to serious complications (for review, see reference 126). RV infection can activate anabolic lipid pathways and subsequently change the levels of phospholipids, sphingolipids, ceramides, and fatty acids (27, 127). FASN inhibition with C75 and TVB-3166 reduced RV replication and production of human RV 16 infectious progeny, respectively (23, 27).

### Pneumoviridae

Respiratory syncytial virus (RSV) belongs to the *Pneumoviridae* family and is an enveloped, negative-sense RNA virus and the leading cause of severe respiratory tract illnesses in infants, the elderly, immunocompromised patients, and adults with comorbidities (128). *In vitro*, RSV-infected lung epithelial cells showed increased formation and dispersion of lipid droplets, and an increase in fatty acid oxidation and fatty acid release, which contributed to the release of pro-inflammatory cytokines (129). In addition, TBV-3166 treatment of RSV-infected A549 cells significantly reduced the production of infectious RSV A and B strain virions, which correlated with reduced palmitate levels (23). TVB-3166 treatment also reduced the levels of viral RNA, F- and G-glycoprotein levels, and prevented viral spread by reducing progeny virion infectivity (23). *In vivo*, TVB-3166 treatment of mice immediately post-infection or 1 day post-infection reduced RSV A (Long) strain titers, reduced lymphocyte infiltration in the lungs, and reduced weight loss in RSV-infected mice (23). In another study, using non-targeted metabolomics of murine lung tissues, it was observed that RSV-infected mice had an increase in free fatty acids, including myristic acid, palmitic acid, oleic acid, and linoleic acid (129). Although the mechanism linking FASN to RSV infection is not completely understood, RSV glycoprotein G (required for cell-surface receptor attachment) and glycoprotein F (required for virion-host membrane fusion) are both palmitoylated (130, 131).

### Sedoreoviridae

Viruses in the family *Sedoreoviridae,* which include rotaviruses, are non-enveloped, double-stranded RNA viruses. Rotaviruses are the most common cause of hospitalization and mortality in children suffering from gastroenteritis (for review, see reference 132). C75 treatment of rotavirus-infected MA104 cells, an African green monkey kidney epithelial cell line, reduced rotavirus infectivity and viral RNA production (29). Rotavirus infection forms non-membranous cytoplasmic structures termed viroplasms used for the initial steps of viral assembly (for review, see reference 133), and it was reported that lipid droplet-associated proteins, a surrogate for lipid droplet detection, co-localized to viroplasms (134). Lipid droplet abrogation disrupted the formation of viroplasms, as well as reduced viral RNA and infectious virion progeny. Finally, rotaviral structural proteins localized with lipid droplet-associated proteins, and the non-structural viral proteins NSP2 and NSP5 were sufficient to recruit lipid droplet components, independent of natural viral infection (134). Thus, it is possible that FASN products are required to produce lipid droplets that support viroplasm formation and viral assembly during rotavirus infection.

### Poxviridae

Vaccinia virus is a double-stranded DNA virus and the prototypic poxvirus. It is closely related to the variola virus, the causative agent of smallpox, and it is used in the smallpox vaccine to induce immunity (135). Vaccinia virus infection of BSC40 cells, an African green monkey-derived kidney cell line, causes FASN to colocalize with the mitochondria and also increases cellular ATP levels (28). C75 treatment of vaccinia-infected cells reduced virion production, viral protein synthesis, and viral assembly and caused a decrease in intracellular viral DNA, and these effects were rescued by the addition of exogenous palmitate. Etomoxir treatment also inhibited viral replication, but inhibitors of protein palmitoylation and phospholipid synthesis did not affect vaccinia virus infection, indicating that FASN activity is mechanistically linked to vaccinia virus replication via ATP production and TCA cycle activation.

### Togaviridae

Members of the *Togaviridae* family, which includes alphaviruses such as chikungunya virus and Mayaro virus, are enveloped, have a positive-sense RNA genome, and are mosquito-borne viruses. Alphavirus infections cause fever, arthralgias and myalgias, fatigue, and rash (136), and they can lead to debilitating, long-lasting fatigue, myositis, and polyarthritis (137). FASN colocalizes with chikungunya virus (CHIKV) replication complexes formed on the host plasma membrane, and a genome-wide siRNA screen identified FASN as a host dependency factor for CHIKV infection (138). In CHIKV-infected HeLa cells, CHIKV infection was decreased by cerulenin treatment, and FASN knockdown reduced both genomic and subgenomic CHIKV RNA synthesis. Similarly, siRNA-mediated FASN knockdown in HEK293T cells reduced CHIKV replication, and FASN inhibition with cerulenin or orlistat treatment reduced CHIKV replication in both 293T and mosquito larvae tissue cells (C6/36) (34). Mayaro virus (MAYV) infection is inhibited by cerulenin and orlistat treatment (34), indicating that FASN is a host dependency factor for at least two alphaviruses. Mechanistically, FASN is required for CHIKV nonstructural protein 1 (nsP1) palmitoylation, which aids in membrane anchoring, and ectopic expression of nsP1 in cells treated with cerulenin, orlistat, or C75 decreased nsP1 membrane association (33). nsP1 mediates membrane anchoring and promotes the formation of viral replication complexes, as *in vitro* nsP1 forms a coat on the outer leaflet of the lipid bilayer of lipid vesicles and deforms their membranes (76). Thus, FASN could supply fatty acids for viral protein acylation and promote the formation of alphavirus replication complexes.

### Retroviridae

Human immunodeficiency virus type-1 (HIV-1) is an enveloped, single-stranded RNA virus and the causative agent of acquired immunodeficiency syndrome (AIDS). In 1988, it was demonstrated that cerulenin treatment of HIV-infected cells inhibited myristoylation and proteolytic cleavage of HIV-1 pr55 Gag, but cerulenin treatment did not affect the processing of the viral glycoprotein gp160 (30). More recently, we reported that HIV-1 infection upregulated FASN protein levels *in vitro,* whereas siRNA-mediated FASN knockdown, or treatment with FASN inhibitors (e.g., Fasnall or C75), reduced virion production (31). FASN knockdown or inhibition did not reduce intracellular levels of HIV-1 Gag protein, indicating that FASN is required for a late-stage process of HIV-1 infection (31). Several HIV-1 proteins are palmitoylated [e.g., Env (77) or myristoylated, e.g., Gag (78, 79), Nef (80)] to facilitate binding to the plasma membrane. Using a bioorthogonal, click-chemistry compatible acetate analog called 5-hexynoic acid (i.e., Alk-4) (139), we observed that FASN products can myristoylate the matrix (MA) domain of HIV-1 Gag protein and that inhibition of FASN with C75 or Fasnall reduced MA myristoylation (32). These studies suggest that HIV-1 leverages FASN activity for viral protein fatty acylation.

### Orthomyxoviridae

Orthomyxoviruses are enveloped, negative-sense, segmented RNA viruses that include influenza A virus (IAV). IAV causes respiratory tract infections characterized by fever, dry cough, sore throat, and runny nose (140). *In vitro*, C75 treatment inhibited IAV replication in Madin-Darby canine kidney epithelial cells (MDCK) (19), and AM580 treatment to block SREBP-1 activity reduced palmitoylation of the hemagglutinin glycoprotein on H1N1 (9). These data suggest that *fasn* transcriptional activation is required for viral protein acylation of the Influenza virus. In addition to the evidence supporting its direct contribution to viral replication as a host dependency factor, our results also suggest that FASN contributes to host antiviral defense mechanisms; specifically, in FASN knockout HAP-1 cells, interferon-beta treatment mediated the inhibition of H1N1 infection was reduced, possibly through the inhibition of interferon-beta induced transmembrane protein 3 (IFITM3) palmitoylation (32).

### Hepadnaviridae

Hepadnaviruses are enveloped, partially double-stranded circular DNA viruses. One family member, hepatitis B virus (HBV), is the primary cause of chronic liver disease in humans (141). *In vitro*, persistent and active HBV infection upregulates FASN expression. HBx, a non-structural, regulatory HBV protein, co-localized with LXR in the nucleus to enhance the binding of LXRa to the SREBP-1 promoter, resulting in the upregulation of SREBP-1 and FASN. In HepG2.2.15.7 cells, a cell line derived from the hepatoblastoma cell line HepG2 that stably expresses HBV, *fasn* is transcriptionally upregulated via SREBP-1, and FASN inhibition with GSK1995010 or knockdown with siRNA decreased extracellular HBV DNA (a surrogate for HBV virions) (17). However, FASN inhibition did not affect intracellular HBV DNA levels and led to an intracellular accumulation of viral nucleocapsid proteins, showing that *in vitro*, FASN plays a role in the late stage of the HBV replication cycle (17). *In vivo*, proteomic analysis of liver tissue from HBV-infected transgenic mice showed HBV-associated upregulation of proteins involved in fatty acid metabolism and synthesis, including FASN (142). Moreover, increased levels of saturated long-chain fatty acids have been reported in people living with either acute or chronic HBV, adenoviral HBV-infected primary rat hepatocytes, and HBV-producing cell lines (for review, see reference 143). The mechanism for FASN during HBV infection remains unclear; however, evidence both *in vitro* and *in vivo* supports FASN as a required host factor for HBV infection.

### Herpesviridae

Herpesviruses are double-stranded DNA viruses comprising three subfamilies: alpha-herpesviruses, beta-herpesviruses, and gamma-herpesviruses. Herpesviruses have two phases: lytic replication and latency. During lytic replication, herpesviruses actively produce infectious progeny virions, whereas during latency, the viral DNA genome is maintained as a circular episome in the nucleus of the infected host with limited viral gene expression (for review, see reference 144). Although the herpesviruses differ in tropism and clinical manifestations, alpha-, beta-, and gamma-herpesviruses have been shown to require FASN and *de novo* FA biosynthesis.

Gamma-herpesviruses include Epstein-Barr virus (EBV) and Kaposi’s sarcoma herpesvirus (KSHV). EBV causes infectious mononucleosis and is associated with endemic Burkitt lymphoma, nasopharyngeal carcinoma, and B-cell lymphomas in immunosuppressed individuals (for review, see reference 145). EBV viral reactivation from latency starts with the expression of the intermediate early proteins BRLF1 (R) and BZLF1 (Z), which serve as transcriptional activators of viral early genes (for review, see reference 146). In several cell types, R protein expression significantly increased *fasn* mRNA and protein levels via the p38 stress MAP kinase pathway (20). FASN expression was required for R to induce activation of Z and subsequent expression of the viral early gene *bmrf*. Cerulenin and C75 treatment of B-cell lines abrogated expression of the viral early gene, showing that this mechanism is FASN-dependent. Thus, FASN expression is an important component of B-cell EBV lytic reactivation, through an unknown mechanism. Of clinical relevance, FASN protein overexpression was also observed in epithelial cells in the lateral tongue of people with oral hairy leukoplakia, which are characteristic white lesions caused by lytic EBV infection (20).

FASN regulation also plays a role during gamma-herpesvirus latency. Separate studies reported that LMP1, the major transforming oncoprotein *in vitro* during EBV latent infection, upregulated FASN levels (21, 147, 148) in both nasopharyngeal carcinoma and B-cells, through different mechanisms. In nasopharyngeal carcinoma, LMP1 increased *fasn* mRNA and protein levels via SREBP-1, in an mTOR-dependent manner (a signaling kinase that can promote lipogenesis) (147), and LMP1-expressing nasopharyngeal cells also increased lipid droplet synthesis. *In vivo*, SREBP-1-mediated transcriptional suppression of lipogenesis inhibited nasopharyngeal carcinoma tumor growth by inducing apoptosis. Clinically, FASN upregulation was correlated with disease progression and poor prognosis of people with nasopharyngeal carcinoma (147).

In B cells, LMP1 post-translationally stabilized FASN, by upregulation of the isopeptidase ubiquitin-specific protease 2 a (USP2a), which prevented FASN degradation through the removal of ubiquitin (21). Subsequently, increased FASN protein levels led to increased fatty acids and lipid droplet formation. *In vitro*, LMP1 transforms primary B cells to immortalized lymphoblastoid cell lines (LCLs). Consequently, LCLs show increased levels of both FASN protein and polyunsaturated fatty acids (21). C75 treatment reduced LMP1-induced FASN protein expression and B-cell transformation, demonstrating the necessity of FASN in the process of EBV-induced B-cell transformation. Additionally, EBV latent infection can induce different types of latency programs that cause cell proliferation and immortalization. Specifically, type III latency cells endogenously express LMP1, whereas Type I latency cells do not (for review, see reference 146). Treatment of cells expressing a type III latency pattern of gene expression with C75 caused more cell death compared with those with a type I latency pattern, showing that LMP1-dependent induction of FASN is important for the survival of EBV-infected, latent cells (21). As a major player in EBV-induced B-cell transformation, FASN could serve as a promising therapeutic target to prevent EBV-dependent B-cell immortalization. Further research is required to better understand the FASN dependency in EBV B-cell latent infection.

KSHV, also known as HHV-8, is the etiological agent of Kaposi’s sarcoma and other lymphoproliferative diseases (for review, see reference 149). A global metabolic screen of endothelial cells latently infected with KSHV identified several dysregulated metabolic pathways, including *de novo* fatty acid synthesis (22). In immortalized microvascular endothelial cell lines, KSHV latent infection increased lipid droplets and long-chain fatty acids, including palmitate and myristate. In a dose-dependent manner, C75 treatment induced apoptosis and death of infected cells, which was partially rescued by the addition of palmitic acid, demonstrating the importance of fatty acid biosynthesis during latent KSHV infection (22). The mechanism linking FASN activity to KSHV replication is unknown.

Alpha-herpesviruses include herpes simplex virus-1 (HSV-1), herpes simplex virus-2, and varicella-zoster virus (VZV). A study from 1986 linked FASN to VZV replication as cerulenin treatment of VZV-infected cells inhibited viral replication and maturation of viral glycoproteins (18). *In vitro,* treatment of HSV-1-infected cells with C75 or EGCG reduced HSV-1 infectious virion production (6), and others have reported that HSV-1 changes host cell lipid biosynthesis (144). Mechanisms linking FASN and alpha-herpesviruses replication remain unidentified.

Beta-herpesviruses include human cytomegalovirus (HCMV) and human herpesviruses 6A, 6B, and 7. HCMV infection upregulates FASN transcription (150), and C75 treatment of HCMV-infected fibroblast cells reduced infectious HCMV virion production, without inducing cell toxicity or apoptosis (19). HCMV infection also increased both the number of lipid droplets (150) and the production of very long-chain fatty acids (151); however, it is unclear if these changes are FASN-dependent. HCMV glycoprotein B can be palmitoylated, which promotes its association with lipid rafts and supports the fusion of HCMV with epithelial cells (81), but it remains unknown if herpesvirus protein fatty acylation occurs via FASN-catalyzed *de novo* fatty acid biosynthesis.

### Astroviridae

Astroviruses are non-enveloped, positive-sense single-stranded RNA viruses primarily transmitted through the fecal-oral route, and astroviruses are associated with gastrointestinal illnesses (152). Astrovirus infection induces membrane rearrangements into double-membrane vesicles, where it is hypothesized that viral assembly occurs (153). In astrovirus-infected colorectal cell lines, astrovirus proteins, viral RNA, FASN protein, and infectious viral particles colocalized to cellular membrane fractions that were enriched in membranes from the Golgi apparatus, endoplasmic reticulum, mitochondria, plasma membrane, and the nucleus (3). siRNA-mediated FASN silencing significantly reduced the synthesis of antigenomic RNA and the structural protein Vp90, as well as the production of infectious astrovirus progeny, demonstrating the requirement of FASN during astrovirus infection, albeit through an unknown mechanism (3).

## CONCLUSIONS

Here, we reviewed 27 different eukaryotic viruses, from 15 different families, whose replication requires FASN-catalyzed *de novo* fatty acid biosynthesis. It is hypothesized that FASN could promote viral replication via three distinct biochemical mechanisms: provision of fatty acids to form lipid droplets that support viral assembly/replication, provision of fatty acids for beta-oxidation and energy production, or provision of fatty acyl groups used in viral (or host) protein acylation (Table 2). For some of these viruses, the downstream uses of FASN-catalyzed fatty acids have been interrogated, including DENV and enterovirus A-71 (i.e., lipid droplet formation and beta-oxidation), vaccinia virus (i.e., beta-oxidation), and CHIKV, SARS-CoV-2, and HIV-1 (i.e., viral protein fatty acylation); however, for most of the viruses discussed herein, experiments probing the biochemical mechanism(s) that link *de novo* fatty acid biosynthesis to viral replication have not been reported. Nevertheless, the fact that at least 27 viruses require the same host metabolic pathway, even if for different mechanistic functions, opens the possibility that *de novo* fatty acid biosynthesis could function as a target for broad-spectrum antiviral therapeutic development.

**TABLE 2:** Classification of viruses on the proposed FASN-supported mechanisms

Mechanism	Virus^*a*^
Beta oxidation	DENV, HCV, vaccinia virus, EV-A71
Lipid droplet formation	DENV, ZIKV, HCV, SARS-CoV-2, MERS-CoV, EV-A71, RSV, rotavirus, EBV, KSHV, HCMV
Protein fatty acylation	HCV, SARS-CoV-2, CHIKV, HIV-1, IAV
Unknown	WNV, CVB3, RV, MAYV, HBV, VZV, HSV-1, astroviruses, SFV, VSV, PIV3, rhinovirus

^*a*^ Viruses are classified based on the presence or absence of evidence for each mechanism.

When considering the role of FASN in viral replication and as a target for antiviral drug development, several potential limitations must also be considered. First, many of the reported associations between FASN and viral replication were established with first-generation FASN inhibitors (e.g., cerulenin, C75), which can have non-specific or off-target effects (101). However, this concern is mitigated, in part, by studies that demonstrate inhibition of viral replication by second and third-generation FASN inhibitors (e.g., HIV-1/Fasnall, RSV/TVB-3166, SARS-CoV-2/TVB-2640, TVB-3664, and TVB-3166). Furthermore, manipulation of cellular FASN levels by gene knockdown or silencing of FASN expression has also demonstrated inhibition of viral replication (e.g., HIV-1, CHIKV, DENV, HCV, HBV, and astroviruses).

Another limitation is the lack of knowledge on the mechanistic role of FASN during viral infection, and several variables warrant further investigation. For example, fatty acid synthesis and fatty acid oxidation are generally considered antagonistic processes. Although the former consumes energy, the latter generates it. In normal cells, these pathways are tightly regulated; however, in cancer cells, both processes can function simultaneously. As viruses reprogram the host lipid metabolism, it is plausible that both processes are manipulated to support viral replication needs. In several viral infections discussed here (Table 2), both lipid droplet production and beta-oxidation appear to be essential. A possible explanation is that FASN activity promotes lipid droplet formation, which supports viral assembly. Lipid droplets are subsequently lysed to provide fatty acids for beta-oxidation and energy production, suggesting a concurrent activation of both metabolic pathways. However, dissecting the mechanistic pathways in the context of FASN and viral replication is challenging. FASN inhibition can lead to the accumulation of malonyl-CoA, which negatively regulates beta-oxidation. Thus, it may be difficult to determine whether reduced viral replication due to FASN inhibition is caused by palmitate depletion or impaired beta-oxidation. Furthermore, certain inhibitors used to study the mechanistic requirements of viral infection, such as etomoxir (a CPT1 inhibitor), exhibit off-target effects that complicate the analysis and experiments to determine the requirement of beta-oxidation in viral replication (154–156). Future studies should employ the use of specific inhibitors or genetic silencing to minimize off-target effects. Another issue to consider when inhibiting FASN activity is the contributions of FASN activity to immune cell activation, proliferation, and survival (104). For example, a previous study identified that FASN inhibition on the KS domain affected the synthesis of acetoacetyl-CoA necessary for cholesterol synthesis and a pro-inflammatory response in macrophages. However, if a domain targeted was downstream of KS, the macrophage pro-inflammatory response was not affected (103). Additional research should clarify the immunological outcomes when FASN is targeted in the context of viral infection. Finally, several groups have knocked out FASN in mice, constitutively (157), conditionally (158), and under tissue-specific conditions (159–161), with pleiotropic effects. Despite these limitations, in humans, one FASN inhibitor (denifanstat) has revealed no treatment-related serious adverse events in the FASCINATE-2 Phase 2b clinical trial and has shown a favorable safety profile in several other studies (94, 97, 98). Thus, host FASN activity can be considered a “druggable” therapeutic target, making FASN an attractive candidate for broad-spectrum antiviral development.
